# Phase II study of magrolimab combined with docetaxel in previously treated metastatic advanced solid tumors

**DOI:** 10.3389/fonc.2026.1786385

**Published:** 2026-04-22

**Authors:** Antoine Italiano, Teresa García Manrique, Enrique Grande Pulido, Katie Kerrigan, Aude Fléchon, Julia Martínez Pérez, Bogdan Żurawski, Muhammad Furqan, Oscar Juan-Vidal, Ulka Vaishampayan, Charlene Fares, Bruno Fang, Brian Vicuna, Laurent Greillier, Vivek Subbiah, Mei Dong, Kai Song, Yiran Zhang, Young Kim, Luis Paz-Ares

**Affiliations:** 1 Département d’oncologie médicale – Sarcomes, Centre Régional de Lutte Contre le Cancer (CLCC) Institut Bergonié, Bordeaux, France; 2 Department of Medical Oncology, Hospital Universitario Virgen Macarena, Seville, Spain; 3 Servicio de Oncología Médica, MD Anderson Cancer Center, Madrid, Spain; 4 Division of Medical Oncology, Huntsman Cancer Institute, University of Utah School of Medicine, Salt Lake City, UT, United States; 5 Department of Medical Oncology, Centre Léon Bérard Centre Régional de Lutte Contre le Cancer, Lyon, France; 6 Medical Oncology Department, Hospital Universitario Virgen del Rocío, Seville, Spain; 7 Department of Outpatient Chemotherapy, Centrum Onkologii Szpital im. prof. Franciszka Łukaszczyka w Bydgoszczy, Bydgoszcz, Poland; 8 Department of Internal Medicine, Holden Comprehensive Cancer Center, University of Iowa Hospitals and Clinics, Iowa City, IA, United States; 9 Medical Oncology Service, Hospital Universitari i Politècnic la Fe, Valencia, Spain; 10 Division of Hematology/Oncology, Department of Internal Medicine, University of Michigan, Ann Arbor, MI, United States; 11 Department of Medical Oncology, Virginia Piper Cancer Center, Allina Health Cancer Institute, Minneapolis, MN, United States; 12 Department of Medical Oncology, Astera Cancer Care, East Brunswick, NJ, United States; 13 Department of Medical Oncology, Comprehensive Cancer Centers of Nevada, Las Vegas, NV, United States; 14 Multidisciplinary Oncology and Therapeutic Innovations Department, Centre d'Essais Précoces en Cancérologie de Marseille (CEPCM), Assistance Publique-Hôpitaux de Marseille (AP-HM), Marseille, France; 15 Department of Investigational Cancer Therapeutics, Division of Cancer Medicine, University of Texas MD Anderson Cancer Center, Houston, TX, United States; 16 Gilead Sciences, Inc., Foster City, CA, United States; 17 Department of Medical Oncology, Hospital Universitario 12 de Octubre, H12O-CNIO Lung Cancer Unit, Complutense University and Ciberonc, Madrid, Spain

**Keywords:** anti-CD47, docetaxel, magrolimab, metastatic non-small cell lung cancer, metastatic small cell lung cancer, metastatic urothelial carcinoma

## Abstract

**Background:**

Novel treatments are needed to improve the poor prognosis of metastatic cancers. The ELEVATE Lung&UC study evaluated magrolimab plus docetaxel in patients with metastatic non-small cell lung cancer (mNSCLC), metastatic small cell lung cancer (mSCLC), and metastatic urothelial carcinoma (mUC).

**Methods:**

This phase II, open-label, multi-arm study enrolled patients who had received 1–2 (mNSCLC, mSCLC) or 2–3 prior lines of therapy (mUC) in the locally advanced/metastatic setting. A safety run-in (SRI) cohort (mNSCLC/mSCLC/mUC) followed by a phase II cohort (three groups: mNSCLC, mSCLC, mUC) were planned. Primary endpoints were incidence of treatment-emergent adverse events (TEAEs; SRI and phase II) and objective response rate (ORR; phase II).

**Results:**

The SRI cohort (n = 9) had no dose-limiting toxicities. In phase II (mNSCLC, 29 patients; mSCLC, 42 patients; mUC, 26 patients), ORRs were 17.2% (mNSCLC), 4.8% (mSCLC), and 3.8% (mUC). Grade ≥3 magrolimab-related TEAE rates were 48.3% (mNSCLC), 47.6% (mSCLC), and 57.7% (mUC). A fatal TEAE suspected as magrolimab related (intracranial hemorrhage) occurred in one patient with mSCLC and brain metastasis (phase II). The study was closed early, which limited the interpretation of results due to short follow-up and limited endpoint maturity.

**Discussion:**

Adding magrolimab to docetaxel had manageable toxicity but no meaningful improvement in efficacy. These results provide insight into the safety and efficacy of anti-CD47–containing therapies and reinforce the need for treatments that address the unmet needs of patients with previously treated metastatic solid tumors.

## 1 Introduction

Platinum-based chemotherapy with/without immune checkpoint inhibitors (ICIs) is standard-of-care for many metastatic solid tumors, including in first-line metastatic non-small cell lung cancer (mNSCLC) or metastatic small cell lung cancer (mSCLC) and in second-line metastatic urothelial cancer (mUC). Disease progression after platinum/ICI treatment leads to a poor prognosis and limited treatment options, with most historically receiving taxane-based chemotherapy (eg, docetaxel) (1–3). In later-lines, taxane clinical activity is modest, with objective response rates (ORRs) of ≈3%–13% (mNSCLC), ≈5%–29% (mSCLC), and ≈11%–14% (mUC) (2, 4, 5). In second-line mUC, enfortumab vedotin (antibody-drug conjugate) has shown improved outcomes (2). Nonetheless, novel treatment options are needed for mNSCLC, mSCLC, and mUC.

A biomarker of interest for many malignancies is the antiphagocytic signal cluster-of-differentiation (CD)-47, which is overexpressed in various tumors, including lung and urothelial (6–8). The monoclonal antibody magrolimab blocks the interaction of CD47 with its receptor, promoting cancer cell phagocytosis (9), and a phase I study showed magrolimab produced objective responses in patients with advanced solid tumors (10). Combining magrolimab with docetaxel may enhance phagocytic cancer cell elimination, as docetaxel can increase prophagocytic signals (eg, calreticulin) on malignant cells (11).

Based on these data, the phase II ELEVATE Lung&UC study evaluated the efficacy/safety of magrolimab plus docetaxel in previously treated metastatic solid tumors.

## 2 Methods

### 2.1 Study design and treatments

This phase II, open-label, multicohort, multicenter study (NCT04827576) consisted of a safety run-in (SRI) cohort followed by a phase II cohort. The SRI enrolled adults with solid tumors who had received 1–3 prior lines (mNSCLC, mSCLC) or 2–3 prior lines (mUC) of therapy in the locally advanced/metastatic setting. Phase II enrolled adults with 1–2 prior lines (mNSCLC, mSCLC) or 2–3 prior lines (mUC) of therapy in the locally advanced/metastatic setting. In phase II, prior treatment with chemotherapy and ICIs was required for mNSCLC and mUC groups; prior chemotherapy with/without immunotherapy was required for the mSCLC group.

Across phase II cohorts, patients who received a taxane within 12 months or were refractory to prior taxane treatment were excluded. Patients with mNSCLC whose tumors had targetable genomic alterations were excluded. Complete eligibility criteria are listed in the **Supplementary Methods**.

The SRI cohort received docetaxel 75 mg/m^2^ intravenously on day (D) 1 of each cycle (C) (21-day cycles) and 1 mg/kg magrolimab (priming dose) on C1D1; 30 mg/kg on C1D8 and C1D15; 30 mg/kg on C2D1, C2D8, and C2D15; and 60 mg/kg on D1 of C3 and beyond. Upon SRI completion, patients were enrolled into phase II, wherein magrolimab was evaluated at the recommended phase II doses (RP2Ds) determined in the SRI.

The study was conducted according to the International Conference on Harmonisation Good Clinical Practice Guidelines, the Declaration of Helsinki and local Institutional Review Board requirements. All patients provided written informed consent before study participation.

### 2.2 Outcomes

A primary endpoint for SRI and phase II cohorts was incidence of adverse events (AEs) and laboratory abnormalities. An additional phase II primary endpoint was investigator-assessed ORR. Phase II secondary endpoints included investigator-assessed progression-free survival (PFS), investigator-assessed duration of response (DOR), and overall survival (OS). The correlation between clinical response and baseline CD47 levels in the biomarker-evaluable population (BEP) was exploratory (**Supplementary Methods**). Endpoints are defined in the **Supplementary Methods**.

### 2.3 Statistical analysis

Planned enrollment included 6–18 patients in the SRI and 96 patients in phase II (mNSCLC, n = 30; mSCLC, n = 40; mUC, n = 26) (**Supplementary Methods**). Dose-limiting toxicities (DLTs) were evaluated during C1 of the SRI phase. DLT incidence was evaluated using patients who had a DLT or who did not have a DLT but who received ≥2 doses of magrolimab and ≥1 dose of docetaxel during the SRI phase. In SRI and phase II cohorts, safety was assessed in all patients who received ≥1 dose of any study drug.

Efficacy analyses were performed in all patients given ≥1 dose of any study drug. The 95% confidence intervals (CIs) for ORR were calculated using the Clopper-Pearson method; PFS, OS, and DOR were estimated by the Kaplan-Meier method with 95% CIs derived using log-log transformation.

## 3 Results

### 3.1 Patients

After magrolimab development in hematologic malignancies was discontinued, the sponsor elected to close this study early (April 2024) as part of a broader strategic reassessment informed by a comprehensive review of all available magrolimab data across multiple solid tumor programs. The decision to close the study was not driven by safety concerns or by the results of a futility analysis.

At data cutoff (22 October 2024), 106 patients were enrolled and treated: nine in the SRI and 97 in phase II (mNSCLC, n = 29; mSCLC, n = 42; mUC, n = 26; **Supplementary Figure 1**). Demographics and baseline characteristics were reflective of the targeted populations (**Table 1**). Most patients had stage IV disease at screening (96.2%) and an Eastern Cooperative Oncology Group performance status of 0–1 (95.3%). Most patients in phase II lung cancer groups were current/former tobacco users (mNSCLC, 62.1%; mSCLC, 59.5%) or current/former smokers (mNSCLC, 82.8%; mSCLC, 95.2%). The most common reason for study discontinuation was death (67.0%) (**Supplementary Figure 1**).

**Table 1:** Patient demographics and baseline disease characteristics.

Characteristic	SRI cohort^1,2^ (n = 9)	Phase II cohort		
		mNSCLC^2^ (n = 29)	mSCLC (n = 42)	mUC (n = 26)
Age, median (range), years	64 (54–78)	66 (36–82)	63 (39–74)	66 (38–78)
Sex, n (%)				
Female	4 (44.4)	6 (20.7)	17 (40.5)	7 (26.9)
Male	5 (55.6)	23 (79.3)	25 (59.5)	19 (73.1)
Race, n (%)				
Asian	1 (11.1)	1 (3.4)	0	1 (3.8)
Black or African American	1 (11.1)	0	1 (2.4)	0
White	7 (77.8)	27 (93.1)	30 (71.4)	19 (73.1)
Other/Not permitted	0	1 (3.4)	11 (26.2)	6 (23.1)
Disease stage at screening, n (%)				
Stage II	0	0	1 (2.4)	0
Stage III	0	1 (3.4)	1 (2.4)	1 (3.8)
Stage IV	9 (100)	28 (96.6)	40 (95.2)	25 (96.2)
ECOG PS, n (%)				
0	1 (11.1)	12 (41.4)	11 (26.2)	7 (26.9)
1	8 (88.9)	15 (51.7)	30 (71.4)	17 (65.4)
2	0	2 (6.9)	1 (2.4)	2 (7.7)
Smoking status, n (%)				
Never	3 (33.3)	4 (13.8)	1 (2.4)	11 (42.3)
Current	2 (22.2)	6 (20.7)	6 (14.3)	6 (23.1)
Former	4 (44.4)	18 (62.1)	34 (81.0)	9 (34.6)
Missing	0	1 (3.4)	1 (2.4)	0
Tobacco use status, n (%)				
Never	6 (66.7)	10 (34.5)	15 (35.7)	14 (53.8)
Current	1 (11.1)	5 (17.2)	3 (7.1)	5 (19.2)
Former	2 (22.2)	13 (44.8)	22 (52.4)	7 (26.9)
Missing	0	1 (3.4)	2 (4.8)	0
Prior lines of anticancer therapy, n (%)				
1	1 (11.1)	13 (44.8)	16 (38.1)	0
2	4 (44.4)	15 (51.7)	23 (54.8)	15 (57.7)
≥3	4 (44.4)	1 (3.4)	3 (7.1)	11 (42.3)
Type of prior anticancer therapy, n (%)				
Chemotherapy	8 (88.9)	29 (100)	42 (100)	26 (100)
Immunotherapy	7 (77.8)	29 (100)	34 (81.0)	26 (100)
Targeted therapy	3 (33.3)	6 (20.7)	3 (7.1)	14 (53.8)
Other	1 (11.1)	1 (3.4)	2 (4.8)	0

Data presented for all enrolled patients.

^1^In the SRI cohort, there were 2 (22.2%), 4 (44.4%), and 3 (33.3%) patients with mNSCLC, mSCLC, and mUC, respectively. ^2^Among 31 patients with NSCLC in the SRI and phase II cohorts, 5 had squamous histology, 19 had adenocarcinoma, and 7 had histology “not otherwise specified.”

ECOG PS, Eastern Cooperative Oncology Group performance status; mNSCLC, metastatic non-small cell lung cancer; mSCLC, metastatic small cell lung cancer; mUC, metastatic urothelial cancer; SRI, safety run-in.

### 3.2 SRI results

**Supplementary Table 1** summarizes treatment-emergent AEs (TEAEs) for the SRI cohort. No DLTs were reported. A fatal TEAE occurred in one patient (gastrointestinal hemorrhage, not considered magrolimab related). Magrolimab RP2Ds were determined to be 30 mg/kg weekly through C2 and 60 mg/kg for C3 and beyond (initial dose level assessed).

### 3.3 Phase II safety

Median (range) duration of magrolimab exposure was 12.7 weeks (0.1–90.3) in the mNSCLC group, 7.6 weeks (0.1–72.1) in the mSCLC group, and 9.1 weeks (1.1–68.1) in the mUC group (**Supplementary Table 2**). Median (range) duration of docetaxel exposure was 7.1 (0.1–69.3), 7.6 (0.1–72.1), and 8.2 (0.1–68.1) weeks, respectively. Common any-grade and grade ≥3 TEAEs are summarized in **Table 2**. The most common grade ≥3 TEAE in all groups was neutropenia (mNSCLC, 48.3%; mSCLC, 38.1%; mUC, 46.2%). Rates of grade ≥3 TEAEs related to magrolimab were 48.3% (mNSCLC), 47.6% (mSCLC), and 57.7% (mUC). **Supplementary Table 3** summarizes TEAEs of clinical importance.

**Table 2:** Most common TEAEs (≥15% incidence).

TEAEs, n (%)	SRI cohort (n = 9)		Phase II cohort							Total (N = 106)	
			mNSCLC (n = 29)		mSCLC (n = 42)		mUC (n = 26)				
	Any grade	Grade ≥3	Any grade	Grade ≥3	Any grade	Grade ≥3		Any grade	Grade ≥3	Any grade	Grade ≥3
Anemia	5 (55.6)	2 (22.2)	22 (75.9)	7 (24.1)	28 (66.7)	14 (33.3)		16 (61.5)	8 (30.8)	71 (67.0)	31 (29.2)
Asthenia	1 (11.1)	1 (11.1)	18 (62.1)	5 (17.2)	23 (54.8)	3 (7.1)		10 (38.5)	2 (7.7)	52 (49.1)	11 (10.4)
Neutropenia^1^	4 (44.4)	4 (44.4)	18 (62.1)	14 (48.3)	19 (45.2)	16 (38.1)		13 (50.0)	12 (46.2)	54 (50.9)	46 (43.4)
Diarrhea	2 (22.2)	1 (11.1)	15 (51.7)	2 (6.9)	19 (45.2)	1 (2.4)		6 (23.1)	0	42 (39.6)	4 (3.8)
Pyrexia	3 (33.3)	0	11 (37.9)	0	8 (19.0)	0		8 (30.8)	0	30 (28.3)	0
Dyspnea	2 (22.2)	0	9 (31.0)	0	15 (35.7)	4 (9.5)		3 (11.5)	0	29 (27.4)	4 (3.8)
Fatigue	6 (66.7)	2 (22.2)	6 (20.7)	2 (6.9)	11 (26.2)	3 (7.1)		6 (22.3)	1 (3.8)	29 (27.4)	8 (7.5)
Decreased appetite	2 (22.2)	0	7 (24.1)	1 (3.4)	17 (40.5)	1 (2.4)		2 (7.7)	0	28 (26.4)	2 (1.9)
Nausea	3 (33.3)	0	5 (17.2)	0	16 (38.1)	0		4 (15.4)	0	28 (26.4)	0
Alopecia	2 (22.2)	0	8 (27.6)	0	6 (14.3)	0		6 (23.1)	0	22 (20.8)	0
Constipation	2 (22.2)	0	6 (20.7)	0	8 (19.0)	0		4 (15.4)	0	20 (18.9)	0
Edema peripheral	3 (33.3)	0	6 (20.7)	0	4 (9.5)	1 (2.4)		5 (19.2)	0	18 (17.0)	1 (0.9)
Vomiting	2 (22.2)	0	4 (13.8)	0	7 (16.7)	0		4 (15.4)	0	17 (16.0)	0
Arthralgia	1 (11.1)	0	6 (20.7)	0	5 (11.9)	0		4 (15.4)	0	16 (15.1)	0

Data presented for patients who received ≥1 dose of any study drug.

AEs were coded according to MedDRA v27.0. Multiple AEs were counted only once per patient for the highest severity grade for each preferred term.

^1^Grouped terms of neutropenia and neutrophil count decreased.

AE, adverse event; MedDRA v27.0, Medical Dictionary for Regulatory Activities version 27.0; mNSCLC, metastatic non-small cell lung cancer; mSCLC, metastatic small cell lung cancer; mUC, metastatic urothelial cancer; SRI, safety run-in; TEAE, treatment-related adverse event.

Rates of TEAEs leading to magrolimab discontinuation were 3.4% (mNSCLC), 11.9% (mSCLC), and 0% (mUC) (**Supplementary Table 1**). Rates of TEAEs leading to magrolimab dose interruption were 37.9% (mNSCLC), 42.9% (mSCLC), and 50.0% (mUC). Five fatal TEAEs occurred: four in the mNSCLC group (hemoptysis, lung abscess, pneumonia, respiratory distress; none magrolimab-related), and one in the mSCLC group (intracranial hemorrhage; suspected as magrolimab-related but confounded by computed tomography-confirmed brain metastasis).

### 3.4 Phase II efficacy

**Figure 1:**
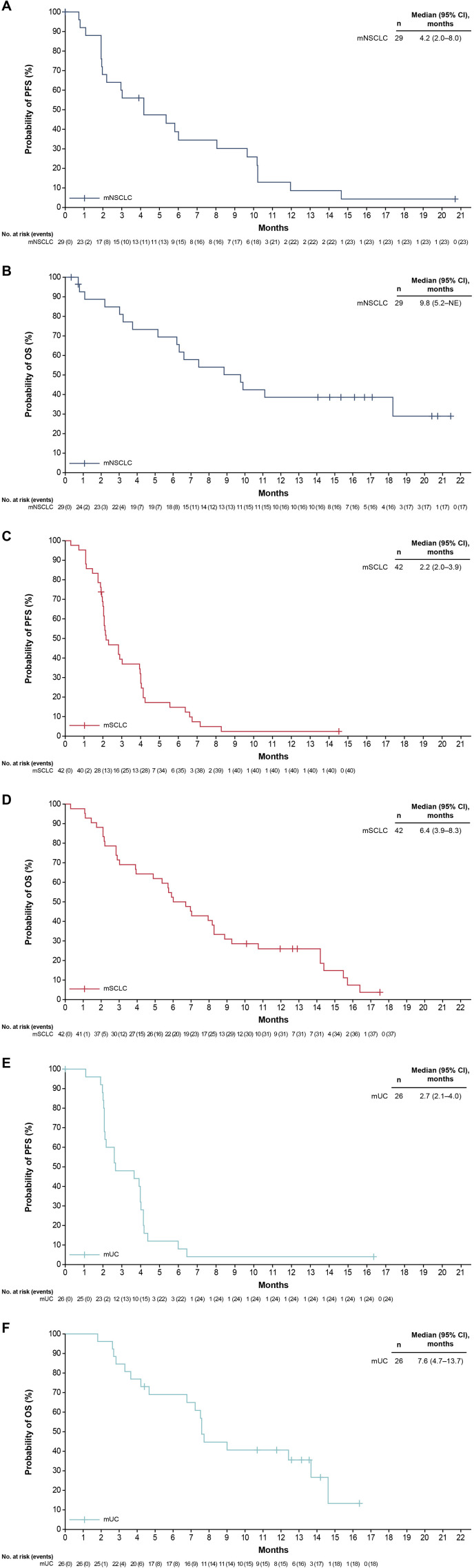
Survival outcomes in phase II efficacy analysis populations. PFS **(A)** and OS **(B)** in the mNSCLC group. PFS **(C)** and OS **(D)** in the mSCLC group. PFS **(E)** and OS **(F)** in the mUC group. CI, confidence interval; mNSCLC, metastatic non-small cell lung cancer; mSCLC, metastatic small cell lung cancer; mUC, metastatic urothelial carcinoma; NE, not evaluable; OS, overall survival; PFS, progression-free survival.

In the mNSCLC group, the ORR (95% CI) was 17.2% (5.8%–35.8%) (**Table 3**); all were partial responses (PRs). Median DOR (95% CI) was 7.6 months (3.7–not estimable [NE]). The disease control rate (DCR) (95% CI) was 55.2% (35.7%–73.6%). At 7.4 months median follow-up for OS, median (95% CI) PFS and OS were 4.2 months (2.0–8.0) (**Figure 1A**) and 9.8 months (5.2–NE) (**Figure 1B**), respectively.

**Table 3:** Response outcomes in phase II.

Outcome	mNSCLC (n = 29)	mSCLC (n = 42)	mUC (n = 26)
ORR^1–3^ [95% CI], %	17.2 [5.8–35.8]	4.8 [0.6–16.2]	3.8 [0.1–19.6]
Best overall response,^1^ n (%)			
CR	0	0	1 (3.8)
PR	5 (17.2)	2 (4.8)	0
SD	11 (37.9)	16 (38.1)	11 (42.3)
PD	5 (17.2)	16 (38.1)	11 (42.3)
Discontinued before first assessment	8 (27.6)	8 (19.0)	3 (11.5)
DCR^2,4^ [95% CI], %	55.2 [35.7–73.6]	42.9 [27.7–59.0]	46.2 [26.6–66.6]
Median time to response^5^ (range), months	2.1 (2.0–6.0)	2.0 (1.9–2.0)	1.6 (1.6–1.6)
Median DOR^2,5,6^ [95% CI], months	7.6 [3.7–NE]	4.7 [4.6–NE]	NE [NE–NE]

Data presented for the efficacy analysis populations.

^1^Confirmed. ^2^Investigator-assessed per RECIST v1.1. ^3^Proportion of patients with a best overall response of confirmed CR or PR. ^4^Proportion of patients who achieve a best overall response of confirmed CR/PR or SD. ^5^Assessed in patients with a best overall response of confirmed CR or PR. ^6^Time from date of first documented CR or PR to the earliest date of documented disease progression or death from any cause, whichever occurs first.

CI, confidence interval; CR, complete response; DCR, disease control rate; DOR, duration of response; mNSCLC, metastatic non-small cell lung cancer; mSCLC, metastatic small cell lung cancer; mUC, metastatic urothelial cancer; NE, not evaluable/estimable; ORR, objective response rate; PD, progressive disease; PR, partial response; RECIST, Response Evaluation Criteria in Solid Tumors version 1.1; SD, stable disease.

In the mSCLC group, the ORR (95% CI) was 4.8% (0.6%–16.2%). Two patients had a PR (DORs, 4.6 and 4.8 months). The DCR (95% CI) was 42.9% (27.7%–59.0%). At 6.4 months median follow-up for OS, median (95% CI) PFS and OS were 2.2 months (2.0–3.9) (**Figure 1C**) and 6.4 months (3.9–8.3) (**Figure 1D**), respectively.

In the mUC group, the ORR (95% CI) was 3.8% (0.1%–19.6%); one patient had a complete response (CR), with a DOR that was NE. The DCR (95% CI) was 46.2% (26.6%–66.6%). At 7.6 months median follow-up for OS, median (95% CI) PFS and OS were 2.7 months (2.1–4.0) (**Figure 1E**) and 7.6 months (4.7–13.7) (**Figure 1F**), respectively.

### 3.5 Exploratory biomarker analyses

CD47 expression was assessed in the BEP of mNSCLC (n = 24) and mUC (n = 20) groups. Baseline characteristics and efficacy between BEP and efficacy analysis populations were similar (data not shown). In the mNSCLC group, responders (CR/PR) may have had higher baseline CD47 expression than nonresponders (stable/progressive disease); PFS was numerically longer for patients with high (CD47 TC ≥35 [median]) versus low CD47-positivity at baseline (median [95% CI] PFS, 8.1 [3.0–10.2] *vs.* 2.2 [0.8–10.2] months) (**Supplementary Figure 2**). For the mUC group, PFS was similar for high (CD47 TC ≥67.5 [median]) versus low CD47 baseline expression (median [95% CI] PFS, 2.4 [1.1–4.0] *vs.* 3.7 [0.3–4.2] months). Most tumor biopsy pairs taken before and after treatment showed increased CD47 expression (**Supplementary Figure 3**).

Cytokine expression was assessed in the BEP of all groups (mNSCLC, mUC: 23 patients each; mSCLC: 21 patients). Magrolimab induced a marked increase of myeloid-related, but not interferon-related, cytokines in blood plasma in all groups 4 hours after magrolimab dosing on C1D8 that returned to baseline at the end of C1 (**Supplementary Figure 4**).

## 4 Discussion

At early study closure, the results suggested magrolimab could be combined with docetaxel with manageable toxicity and no DLTs but limited efficacy. The magrolimab RP2Ds were 30 mg/kg weekly through C2 and 60 mg/kg for C3 and beyond. At these doses, the types of TEAEs observed with combination treatment were consistent with those observed for magrolimab in other malignancies (10, 12–21) and single-agent taxanes in mNSCLC, mSCLC, and mUC (2, 4, 5, 22). Early closure led to a shorter follow-up than intended and limited endpoint maturity (especially for OS); thus, the study results should be interpreted with caution. The clinical profile of single-agent docetaxel has been well characterized in mNSCLC pivotal studies (23, 24). Anemia, neutropenia, and infections are among the most commonly reported AEs for docetaxel. These are also AEs of clinical interest for magrolimab, with anemia being an on-target effect (25). Treatment-emergent grade ≥3 anemia and infection rates we observed with magrolimab plus docetaxel (mNSCLC group) were numerically higher than those previously reported for single-agent docetaxel (anemia: 24% *vs.* 9%; infections: 17% *vs.* 10%), whereas the grade ≥3 neutropenia rate was numerically lower (48% *vs.* 65%) (23). The rate of study discontinuations due to TEAEs with the combination was similar to that previously reported for docetaxel alone (14% *vs.* 19%) (24). However, caution when making cross-trial comparisons is advised because of different study designs, dosing regimens, and populations.

At study closure, the highest ORR and longest PFS and OS were observed in the mNSCLC group; limited activity was seen in the mSCLC and mUC groups (ORR <5%). Response and survival results observed with combination therapy herein were within the range previously reported for single-agent docetaxel in second-line mNSCLC (ORR, 17% *vs.* 3%–13%; median PFS, 4.2 *vs.* 3.0–4.5 months; median OS, 9.8 *vs.* 5.7–11.3 months (3, 4, 24)) and in second-line mUC (ORR, 4% *vs.* 13%–14%; median PFS, 2.7 *vs.* 1.6–4.0 months; median OS, 7.6 *vs.* 7.0–9.0 months (2)). In second-line or later mSCLC, magrolimab plus docetaxel efficacy outcomes were within range of those reported previously for single-agent (nab)paclitaxel with different regimens (weekly, triweekly) (ORR, 5% *vs.* 5%–29%; median PFS, 2.2 *vs.* 2.0–2.5 months; median OS, 6.4 *vs.* 3.3–5.9 months) (5). With the caveat that cross-trial comparisons should be interpreted with caution, limited benefit was observed with magrolimab plus docetaxel versus single-agent chemotherapy in these pretreated populations.

Development of anti-CD47 therapies has been challenging thus far, spurring further investigation into mechanisms that could affect anti-CD47 efficacy. Although small sample sizes and early closure limit interpretation of our results, biomarker analyses indicated that although magrolimab induced myeloid-related cytokine activity, it did not trigger a sufficient T-cell response to provide an efficacy benefit in the solid tumors evaluated. Based on this, evaluation of combination strategies that incorporate anti-CD47 therapies with agents that more directly promote T-cell function may be of interest. Studies have also shown that engagement of activating FcγR signaling during anti-CD47 treatment enhances T-cell response, and Fc-optimized anti-CD47 therapies are under investigation (26, 27). Beyond the insufficient T-cell response, there are other factors that may explain the limited efficacy benefit observed for the addition of magrolimab to docetaxel. CD47 is not the only antiphagocytic signal in the solid tumor microenvironment, and blockade of other immune checkpoints may be needed for adequate phagocytic elimination of cancer cells (28, 29). Additionally, nearly all patients had received prior immunotherapy, and the study population may have been enriched with immunotherapy-resistant phenotypes (30).

Despite these challenges, CD47 remains a target of interest for metastatic solid tumors. Previous studies have shown that CD47 expression correlates with metastasis, clinical stage, and poor prognosis in NSCLC (6). Our biomarker data showed that CD47 expression in pretreatment tumor biopsies had a trending correlation with best overall response and PFS in mNSCLC after magrolimab plus docetaxel treatment. Real-world data previously showed that higher pretreatment CD47 mRNA was associated with improved outcomes in chemotherapy-treated mNSCLC (31). Taken together, these magrolimab- and chemotherapy-specific correlational data support CD47 as a prognostic factor in mNSCLC. However, this interpretation should be viewed as hypothesis generating only, as the biomarker analyses were exploratory and limited by sample size. Our findings should be confirmed in larger evaluations of baseline tumor CD47 expression as a potential predictive or prognostic biomarker in future NSCLC trials of CD47-targeted agents. As a target, CD47 remains under investigation, with multiple clinical trials examining other anti-CD47–based therapies in lung and urothelial cancers (eg, lung: NCT05780307, NCT05403554; UC: NCT05524545).

Despite the termination of magrolimab development, our results provide insights into the efficacy/safety of anti-CD47 therapy plus docetaxel in metastatic lung and urothelial cancers. Although the addition of magrolimab to docetaxel had manageable toxicity, it did not demonstrate a meaningful efficacy improvement compared with historical outcomes for standard chemotherapy. These results contribute to the understanding of CD47 blockade, may inform the development of other anti-CD47 agents for solid tumors, and highlight the continued need for novel therapies/drug targets for metastatic cancers.

## Data Availability

Gilead Sciences shares anonymized individual patient data upon request or as required by law or regulation with qualified external researchers based on submitted curriculum vitae and reflecting non-conflict of interest. The request proposal must also include a statistician. Approval of such requests is at Gilead Sciences’ discretion and is dependent on the nature of the request, the merit of the research proposed, the availability of the data, and the intended use of the data. Requests to access the datasets should be directed to datarequest@gilead.com.
