# Supplementary material for: Phase II study of magrolimab combined with docetaxel in previously treated metastatic advanced solid tumors
Source: Front Oncol. 2026 Apr 22;16:1786385. doi: 10.3389/fonc.2026.1786385 (PMC13143664; doi:10.3389/fonc.2026.1786385)
Supplement: Supplementary file 1 [file DataSheet1.docx]

### Phase II study of magrolimab combined with docetaxel in previously treated metastatic advanced solid tumors

### Antoine Italiano, Teresa García Manrique, Enrique Grande Pulido, et al.

## Supplementary material

## Supplementary methods

## *Inclusion criteria*

All patients were required to meet all of the following inclusion criteria to be eligible for participation in the study:

1. Provided informed consent
2. Were willing and able to comply with clinic visits and procedures outlined in the study protocol
3. Male or female age ≥18 years
4. Eastern Cooperative Oncology Group performance status of ≤2
5. Laboratory measurements, blood counts:
   1. Hemoglobin must be ≥9 g/dL before the initial dose of study drug
   2. Absolute neutrophil count ≥1.5 × 10^9/^L
   3. Platelets ≥100 × 10^9^/L
6. Laboratory measurements, renal function:
   1. Adequate renal function as demonstrated by a creatinine clearance of ≥30 mL/min; calculated by the Cockcroft Gault formula.
7. Adequate liver function, as demonstrated by:
   1. Aspartate aminotransferase ≤2.5 × upper limit of normal (ULN) or ≤5 × ULN in patients with liver metastases
   2. Alanine aminotransferase ≤2.5 × ULN or ≤5 × ULN in patients with liver metastases
   3. Bilirubin ≤1.5 × ULN or ≤3.0 × ULN and primarily unconjugated if patient had a documented history of Gilbert syndrome or genetic equivalent
8. Pretreatment blood cross-match completed
9. Male and female patients of childbearing potential who engage in heterosexual intercourse must have agreed to use protocol-specified method(s) of contraception
10. Measurable disease according to Response Evaluation Criteria in Solid Tumors (RECIST) v1.1. Previously irradiated lesions were considered as measurable disease only if disease progression had been unequivocally documented at that site since radiation

In addition to meeting the inclusion criteria for all patients, the following cohort-specific inclusion criteria were required:

Safety Run-in (SRI) cohort

1. Patients with metastatic advanced solid tumors who had ≥1 prior line of systemic anticancer therapy (metastatic non-small cell lung cancer [mNSCLC], metastatic small cell lung cancer [mSCLC]) in a locally advanced/metastatic setting, or 2 prior lines of systemic anticancer therapy (metastatic urothelial cancer [mUC]) in a locally advanced/metastatic setting, and not >3 prior lines of systemic anticancer therapy in a locally advanced/metastatic setting

Phase II cohort

1. mNSCLC group: Patients with mNSCLC who had treatment with platinum-based chemotherapy and immune checkpoint inhibitor therapy in a locally advanced/metastatic setting, either in combination or sequentially (unless not eligible for one of these therapies) were eligible. At least 1 prior line of systemic anticancer therapy in a locally advanced/metastatic setting was required and not >2 prior lines of systemic anticancer therapy in a locally advanced/metastatic setting were allowed
   1. Patients treated with a taxane within 12 months or refractory to prior taxane treatment were excluded
   2. Patients whose tumors had genomic alterations for which there are approved therapies (eg, *EGFR, ROS1, ALK, NTRK,* MET exon 14) were excluded
2. mSCLC group: Patients with mSCLC who had prior treatment with platinum-based chemotherapy with or without immunotherapy are eligible. At least 1 prior line of systemic anticancer therapy in a locally advanced/metastatic setting was required and not >2 prior lines of systemic anticancer therapy in a locally advanced/metastatic setting were allowed
   1. Patients treated with a taxane within 12 months or refractory to prior taxane treatment were excluded
3. mUC group: Patients with mUC who had prior treatment with systemic chemotherapy and immune checkpoint inhibitor therapy in a locally advanced/metastatic setting (unless not eligible for one of these therapies) were eligible. At least 2 prior lines of systemic anticancer therapy in a locally advanced/metastatic setting were required and not >3 prior lines of systemic anticancer therapy in a locally advanced/metastatic setting were allowed
   1. Patients treated with a taxane within 12 months or refractory to prior taxane treatment were excluded

#### Exclusion criteria

Patients who met any of the following exclusion criteria were not allowed to enroll:

1. Positive serum pregnancy test
2. Breastfeeding female
3. Active central nervous system (CNS) disease. Patients with asymptomatic and stable, treated CNS lesions (radiation and/or surgery and/or other CNS-directed therapy who were off corticosteroids for ≥4 weeks) were allowed
4. Red blood cell (RBC) transfusion dependence, defined as requiring >2 units of packed RBC transfusions during the 4-week period before screening. RBC transfusions were permitted during the screening period and before enrollment to meet the hemoglobin inclusion criteria
5. History of hemolytic anemia, autoimmune thrombocytopenia or Evans syndrome in the past 3 months
6. Known hypersensitivity to any of the study drugs, the metabolites, or formulation excipient
7. Prior treatment with cluster-of-differentiation (CD) 47 or signal regulatory protein α-targeting agents
8. Concurrent participation in another interventional clinical study
9. Known inherited or acquired bleeding disorders
10. Significant disease or medical conditions, as assessed by the investigator and sponsor, that would substantially increase the risk-benefit ratio of participating in the study, included acute myocardial infarction within the past 6 months, unstable angina, uncontrolled diabetes mellitus, significant active infections, and congestive heart failure New York Heart Association Class III–IV
11. Second malignancy, except treated basal cell or localized squamous skin carcinomas, localized prostate cancer, or other malignancies for which patients were not on active anticancer therapies and who were in complete remission for >3 years
12. Known active or chronic hepatitis B or C infection or HIV infection in medical history at the time of study participation
13. Prior anticancer therapy including but not limited to chemotherapy, immunotherapy, or investigational agents within 4 weeks before magrolimab was not permitted

#### Dose-limiting toxicities (DLTs)

All toxicities were graded according to the National Cancer Institute Common Terminology Criteria for Adverse Events (AEs) v5.0. The DLT evaluable population included all SRI-enrolled patients who either had a DLT any time after initiation of the first magrolimab infusion or who did not have a DLT and completed ≥2 magrolimab infusions and ≥1 dose of docetaxel.

A DLT was defined as any:

- Grade ≥3 hematologic toxicity
- Event meeting Hy’s Law criteria
- Grade ≥3 nonhematologic toxicity that had worsened in severity from pretreatment baseline during the DLT assessment period
- In the opinion of the investigator, the treatment-emergent AE (TEAE) was at least possibly related to magrolimab

The following were exceptions to the DLT definition and were not considered a DLT:

- Grade 3 anemia; however, grade 3 hemolytic anemia that was medically significant, required hospitalization or prolonged existing hospitalization, or led to disabling or limiting self-care activities of daily living was considered a DLT
- Grade 3 febrile neutropenia that had responded clinically within 72 hours of maximal supportive care
- Grade 3 neutropenia that resolved to grade 2 or pretreatment baseline with supportive care (including growth factors) within 14 days
- Grade 3 indirect/unconjugated hyperbilirubinemia that resolved to grade ≤2 with supportive care within 1 week and was not associated with other clinically significant consequences.
- Grade 3 electrolyte abnormalities that improved to grade ≤2 or baseline within 72 hours, were not clinically complicated, and resolved spontaneously or responded to conventional medical interventions
- Grade 3 elevation in alanine aminotransferase, aspartate aminotransferase, or alkaline phosphatase that resolved to grade ≤2 with supportive care within 1 week and was not associated with other clinically significant consequences
- Grade 3 nausea/vomiting or diarrhea that resolved to grade ≤2 within 72 hours with adequate antiemetic and other supportive care
- Grade 3 fatigue that resolved to grade ≤2 within 1 week on study
- Grade 3 magrolimab and docetaxel infusion reactions in the absence of an optimal pretreatment regimen, which was defined as acetaminophen or a comparable nonsteroidal anti-inflammatory agent, plus an antihistamine and corticosteroids
- Grade 3 tumor lysis syndrome or electrolyte disturbances (hyperkaliemia, hypophosphatemia, hyperuricemia) that resolved to grade ≤2 or baseline within 72 hours
- Grade 3 or 4 lymphopenia or leukopenia not associated with other clinically significant consequences
- Transient (≤48 hours) grade 3 local reactions, flu-like symptoms, myalgias, fever, headache, acute pain, or skin toxicity that resolved to grade ≤2 within 72 hours after medical management (eg, supportive care, including immunosuppressant treatment) had been initiated

#### Endpoint definitions

The primary endpoint of incidence of TEAEs and laboratory abnormalities were classified according to the National Cancer Institute Common Terminology Criteria for AEs v5.0. The objective response rate was defined as the proportion of patients who had a complete or partial response per RECIST v1.1. Progression-free survival was defined as the time from the date of first dose until disease progression per RECIST v1.1 or death from any cause, whichever occurred first. Duration of response was defined as the time from first documentation of complete or partial response to earliest documented date of disease progression per RECIST v1.1 or death from any cause, whichever occurred first. Overall survival was defined as the date of first dose to death from any cause.

#### Biomarker analysis

Biomarker analysis was conducted in patients enrolled in either the SRI or phase II cohorts who received any study drug and had ≥1 evaluable biomarker measurement available (biomarker-evaluable population); data were pooled. CD47 expression was measured by EPR21794 clone on the membrane of tumor cells by immunohistochemistry (IHC) in patients with mNSCLC and mUC; the mSCLC samples collected (cell aspirate) were unsuitable for IHC staining. The cutoff to define CD47 low versus CD47 high (percent positive) was based on the median value of all baseline tumor samples within each cancer type. Cytokines were quantified from patient plasma using the ELLA platform in-house. Significance was assessed by comparing cytokine data obtained before and 4 hours after magrolimab treatment on cycle 1, day 8 using a paired Wilcoxon test, and P-values adjusted by false discovery rate correction.

#### Statistical analysis

For the phase II cohort, a sample size of 30 patients in the mNSCLC group and 40 patients in the mSCLC group were calculated by 1 sample portion test, while the sample size of 26 patients in the mUC group was calculated using exact methods because of a smaller sample size. Sample sizes were calculated to provide 81 %, 84 %, and 78 % power at a 1-sided α of 0.2, 0.15, and 0.1, respectively. The null overall response rate for each respective tumor group was based on historical efficacy data for taxanes in the second-line setting [1–3]. Power calculations were performed using EAST 6.5 and nQuery 8.0.

#### Supplementary references

1. J. Bellmunt, R. de Wit, D.J. Vaughn, et al., Pembrolizumab as second-line therapy for advanced urothelial carcinoma, N. Engl. J. Med*.* 376 (11) (2017) 1015-1026. doi: 10.1056/NEJMoa1613683.
2. A. Rittmeyer, F. Barlesi, D. Waterkamp, et al., Atezolizumab versus docetaxel in patients with previously treated non-small-cell lung cancer (OAK): a phase 3, open-label, multicentre randomised controlled trial, Lancet. 389 (10066) (2017) 255-265. doi: 10.1016/S0140-6736(16)32517-X.
3. E.F. Smit, E. Fokkema, B. Biesma, et al., A phase II study of paclitaxel in heavily pretreated patients with small-cell lung cancer, Br. J. Cancer. 77 (2) (1998) 347-351. doi: 10.1038/bjc.1998.54.

#### Supplementary tables

**Table S1.** TEAE summary.

| **n (%)** | **SRI cohort**  **(n = 9)** | **Phase II cohort** | | | **Total**  **(N = 106)** |
| --- | --- | --- | --- | --- | --- |
|  |  | **mNSCLC**  **(n = 29)** | **mSCLC**  **(n = 42)** | **mUC**  **(n = 26)** |  |
| Any-grade TEAEs | 9 (100) | 29 (100) | 42 (100) | 26 (100) | 106 (100) |
| Related to any study drug | 9 (100) | 29 (100) | 42 (100) | 25 (96.2) | 105 (99.1) |
| Related to magrolimab | 7 (77.8) | 27 (93.1) | 37 (88.1) | 21 (80.8) | 92 (86.8) |
| Grade ≥3 TEAEs | 8 (88.9) | 22 (75.9) | 34 (81.0) | 24 (92.3) | 88 (83.0) |
| Related to any study drug | 8 (88.9) | 21 (72.4) | 30 (71.4) | 22 (84.6) | 81 (76.4) |
| Related to magrolimab | 4 (44.4) | 14 (48.3) | 20 (47.6) | 15 (57.7) | 53 (50.0) |
| Serious TEAEs | 5 (55.6) | 16 (55.2) | 18 (42.9) | 12 (46.2) | 51 (48.1) |
| Related to any study drug | 2 (22.2) | 10 (34.5) | 12 (28.6) | 8 (30.8) | 32 (30.2) |
| Related to magrolimab | 2 (22.2) | 6 (20.7) | 8 (19.0) | 4 (15.4) | 20 (18.9) |
| TEAEs leading to discontinuation of any study drug | 1 (11.1) | 8 (27.6) | 8 (19.0) | 0 | 17 (16.0) |
| Leading to discontinuation of magrolimab | 0 | 1 (3.4) | 5 (11.9) | 0 | 6 (5.7) |
| TEAEs leading to dose interruption of any study drug | 5 (55.6) | 12 (41.4) | 19 (45.2) | 14 (53.8) | 50 (47.2) |
| Leading to dose interruption of magrolimab | 5 (55.6) | 11 (37.9) | 18 (42.9) | 13 (50.0) | 47 (44.3) |
| Fatal TEAEs | 1 (11.1)^1^ | 4 (13.8)^2^ | 1 (2.4)^3^ | 0 | 6 (5.7) |
| Related to magrolimab | 0 | 0 | 1 (2.4) | 0 | 1 (0.9) |

Data presented for all enrolled patients.

AEs were coded according to MedDRA v27.0. Multiple AEs were counted only once per patient for the highest severity grade for each system organ class and preferred term.

^1^Due to gastrointestinal hemorrhage. ^2^Due to hemoptysis, lung abscess, pneumonia, and respiratory distress (n = 1 each). ^3^Due to intracranial hemorrhage.

AE, adverse event; MedDRA v27.0, Medical Dictionary for Regulatory Activities version 27.0; mNSCLC, metastatic non-small cell lung cancer; mSCLC, metastatic small cell lung cancer; mUC, metastatic urothelial cancer; SRI, safety run-in; TEAE, treatment-emergent adverse event.

##### Table S2. Duration of exposure to magrolimab and docetaxel.

| **Median (range)** | **SRI cohort**  **(n = 9)** | **Phase II cohorts** | | |
| --- | --- | --- | --- | --- |
|  |  | **mNSCLC**  **(n = 29)** | **mSCLC**  **(n = 42)** | **mUC**  **(n = 26)** |
| Magrolimab |  |  |  |  |
| Duration of exposure, weeks | 12.6 (0.1–113.3) | 12.7 (0.1–90.3) | 7.6 (0.1–72.1) | 9.1 (1.1–68.1) |
| Cycles of exposure | 3.0 (1.0–37.0) | 4.0 (1.0–31.0) | 3.0 (1.0–25.0) | 3.5 (1.0–22.0) |
| Docetaxel |  |  |  |  |
| Duration of exposure, weeks | 10.4 (0.1–113.3) | 7.1 (0.1–69.3) | 7.6 (0.1–72.1) | 8.2 (0.1–68.1) |
| Cycles of exposure | 3.0 (1.0–37.0) | 4.0 (1.0–25.0) | 3.0 (1.0–25.0) | 3.0 (1.0–22.0) |

mNSCLC, metastatic non-small cell lung cancer; mSCLC, metastatic small cell lung cancer; mUC, metastatic urothelial cancer; SRI, safety run-in.

##### Table S3. Summary of TEAEs of clinical importance.

| **n (%)** | **SRI cohort**  **(n = 9)** | **Phase II cohort** | | | **Total**  **(N = 106)** |
| --- | --- | --- | --- | --- | --- |
|  |  | **mNSCLC**  **(n = 29)** | **mSCLC**  **(n = 42)** | **mUC**  **(n = 26)** |  |
| Number of patients with any AE of clinical importance | 6 (66.7) | 28 (96.6) | 36 (85.7) | 24 (92.3) | 94 (88.7) |
| Anemia^1^ | 5 (55.6) | 22 (75.9) | 29 (69.0) | 17 (65.4) | 73 (68.9) |
| Infusion related reaction^2^ | 1 (11.1) | 7 (24.1) | 7 (16.7) | 3 (11.5) | 18 (17.0) |
| Myocardial infarction | 0 | 0 | 1 (2.4) | 0 | 1 (0.9) |
| Pneumonitis | 0 | 2 (6.9) | 2 (4.8) | 0 | 4 (3.8) |
| Serious infections^3^ | 1 (11.1) | 5 (17.2) | 7 (16.7) | 5 (19.2) | 18 (17.0) |
| Severe neutropenia^4^ | 4 (44.4) | 14 (48.3) | 17 (40.5) | 15 (57.7) | 50 (47.2) |
| Thromboembolic events^5^ | 2 (22.2) | 4 (13.8) | 4 (9.5) | 3 (11.5) | 13 (12.3) |
| Transfusion reactions due to magrolimab interference with red blood cell typing | 0 | 2 (6.9) | 0 | 0 | 2 (1.9) |

AEs were coded according to MedDRA v27.0. Multiple AEs were counted only once per patient for the highest severity grade for each preferred term.

Data are presented for safety analysis populations.

^1^Includes preferred terms of anemia, hyperbilirubinemia, and blood bilirubin increase. ^2^Includes preferred terms of infusion-related reaction and rash. ^3^Includes preferred terms of pneumonia, sepsis, respiratory tract infection, bronchitis, *Clostridium difficile* infection, encephalitis, kidney infection, lung abscess, neutropenic sepsis, *Pneumocystis jirovecii* pneumonia, pneumonia viral, and urinary tract infection. ^4^Includes preferred terms of neutropenia, febrile neutropenia, and neutrophil count decreased. ^5^Includes preferred terms of deep vein thrombosis, pulmonary embolism, superficial vein thrombosis, atrial thrombosis, myocardial infarction, and vascular device occlusion.

AE, adverse event; MedDRA, Medical Dictionary for Regulatory Activities version 27.0; mNSCLC, metastatic non-small cell lung cancer; mSCLC, metastatic small cell lung cancer; mUC, metastatic urothelial cancer; SRI, safety run-in; TEAE, treatment-emergent adverse event.

#### Supplementary Figures

##### Figure S1. ELEVATE Lung&UC CONSORT diagram. mNSCLC, metastatic non-small cell lung cancer; mSCLC, metastatic small cell lung cancer; mUC, metastatic urothelial carcinoma; SRI, safety run-in.


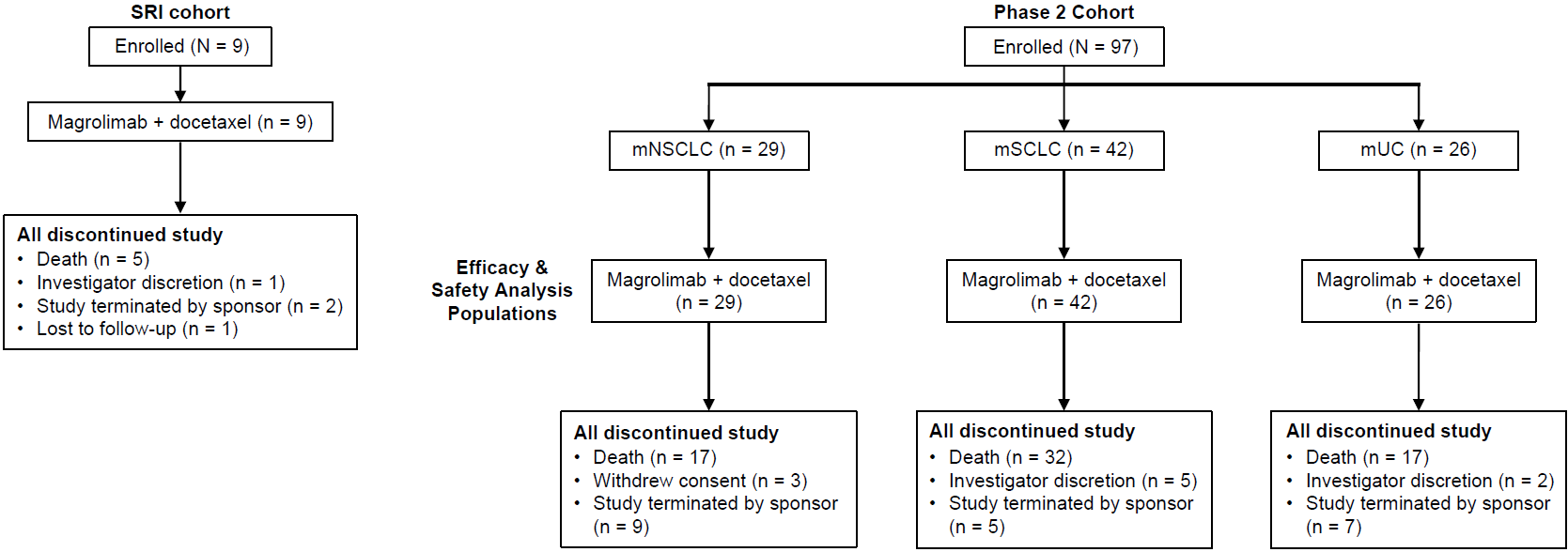


##### Figure S2. Association between tumor cell membrane CD47 expression (measured as percent positive) at baseline and best overall response (A) and PFS after magrolimab + docetaxel treatment in the BEP (B). Subjects in (B) are divided into two groups by median CD47 expression within each tumor group. BEP, biomarker-evaluable population; CD, cluster-of-differentiation; CI, confidence interval; CR/PR, complete or partial response; mNSCLC, metastatic non-small cell lung cancer; mUC, metastatic urothelial cancer; PFS, progression-free survival; SD/PD, stable or progressive disease; TC, tumor cell.

**
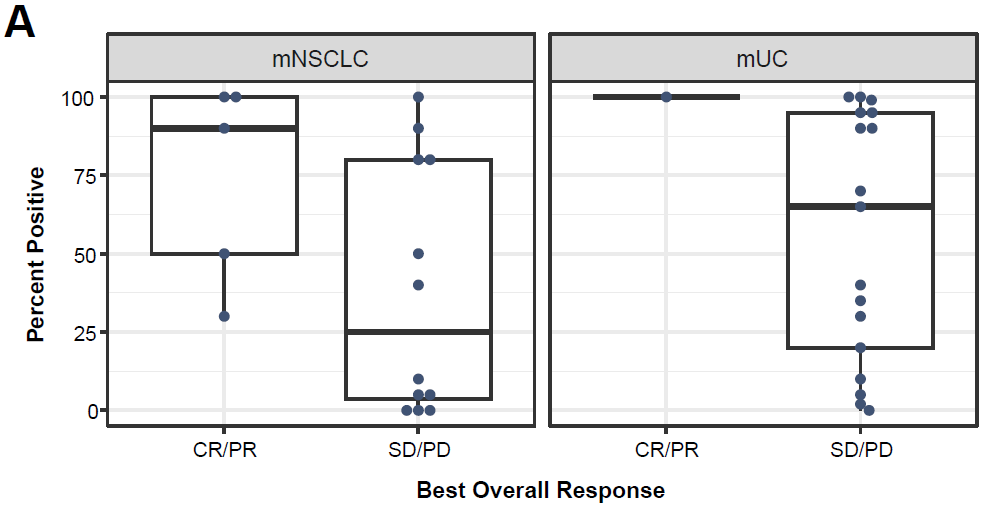
**

**
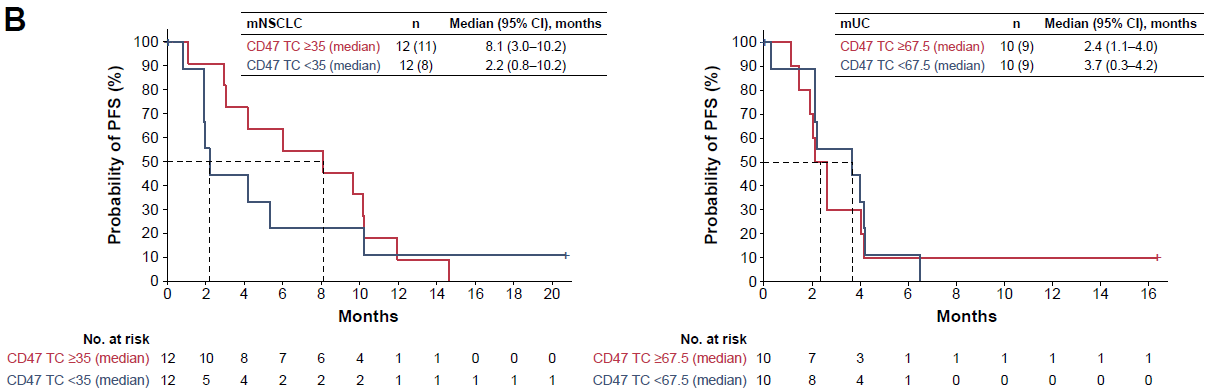
**

**Figure S3.** Tumor cell membrane CD47 expression (measured as H-score) in tumor biopsies taken before and after magrolimab + docetaxel treatment in the BEP. BEP, biomarker-evaluable population; C, cycle; CD, cluster-of-differentiation; CR/PR, complete or partial response; D, day; mNSCLC, metastatic non-small cell lung cancer; mUC, metastatic urothelial cancer; SCR, screening; SD/PD, stable or progressive disease.


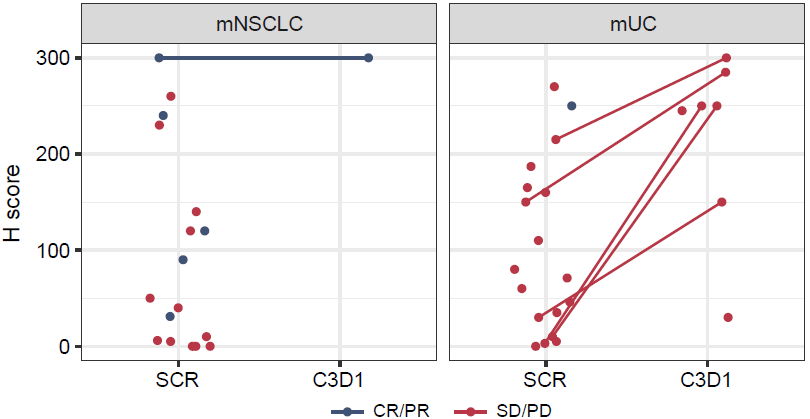


##### Figure S4. Effect of magrolimab + docetaxel treatment on cytokine levels in the BEP. **P* < 0.05; ***P* < 0.01; ****P* < 0.001; *****P* < 0.0001*.* 4HR, 4 hours after treatment; BEP, biomarker-evaluable population; C, cycle; CCL, chemokine ligand; CXCL, chemokine (C-X-C motif) ligand; D, day; IFN, interferon; IL, interleukin; IQR, interquartile range; Magro, magrolimab; MCP, monocyte chemoattractant; MIG, monokine induced by γ-interferon; MIP, macrophage inflammatory protein; mNSCLC, metastatic non-small cell lung cancer; mSCLC, metastatic small cell lung cancer; mUC, metastatic urothelial cancer; ns, not significant; PRE, pretreatment; TNF, tumor necrosis factor.

**
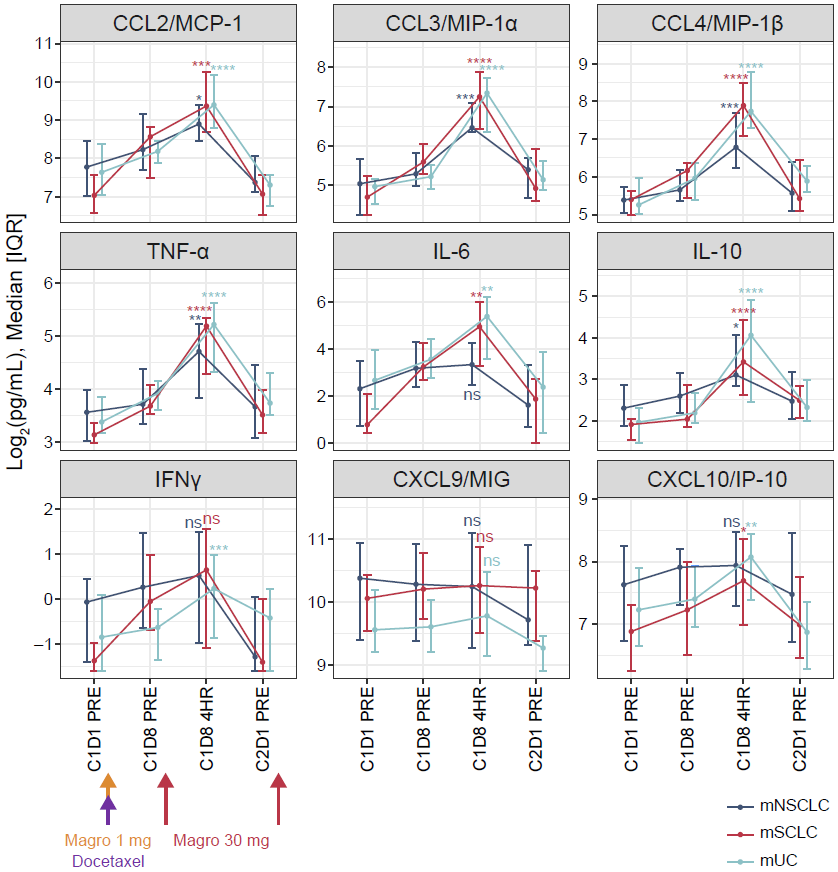
**
